# Ruxolitinib is effective in the treatment of a patient with refractory T‐ALL

**DOI:** 10.1002/jha2.143

**Published:** 2020-12-04

**Authors:** Sonia Jaramillo, Hannah Hennemann, Peter Horak, Veronica Teleanu, Christoph E. Heilig, Barbara Hutter, Albrecht Stenzinger, Hanno Glimm, Benjamin Goeppert, Carsten Müller‐Tidow, Stefan Fröhling, Stefan Schönland, Richard F. Schlenk

**Affiliations:** ^1^ Department of Hematology Oncology, and Rheumatology Heidelberg University Hospital University of Heidelberg Heidelberg Germany; ^2^ Division of Translational Medical Oncology National Center for Tumor Diseases (NCT) Heidelberg, and German Cancer Research Center (DKFZ) Heidelberg Germany; ^3^ Computational Oncology, Molecular Diagnostics Program NCT and DKFZ Heidelberg Germany; ^4^ Division of Applied Bioinformatics DKFZ Heidelberg Germany; ^5^ Institute of Pathology University of Heidelberg Heidelberg Germany; ^6^ German Cancer Consortium (DKTK) Heidelberg Germany; ^7^ Department of Translational Medical Oncology NCT Dresden, Dresden, and DKFZ Heidelberg Germany; ^8^ Center for Personalized Oncology NCT Dresden and University Hospital Carl Gustav Carus Dresden Technical University of Dresden Dresden Germany; ^9^ Translational Functional Cancer Genomics NCT and DKFZ Heidelberg Germany; ^10^ NCT‐Trial Center NCT Heidelberg DKFZ and Heidelberg University Hospital Heidelberg Germany

## Abstract

T‐cell acute lymphoblastic leukemia (T‐ALL) is a rare, aggressive T‐cell malignancy. Chemotherapy alone cures only 25‐45% of the cases, thus, novel treatment agents and strategies are urgently needed. We assessed the efficacy of ruxolitinib in a patient with a cutaneous relapse after allogeneic blood cell transplantation of a refractory T‐ALL with a Janus kinase 3 (*JAK3*) mutation. In this case report, we were able to show the potential benefit of the JAK inhibitor ruxolitinib in *JAK3*‐mutated refractory T‐ALL and emphasize the importance of integrating molecular markers in current treatment decision making for patients with T‐ALL.

## 1 CASE PRESENTATION

In 2014, a 56‐year‐old female with a history of lobular breast carcinoma with invasive ductal component, hormone‐receptor–positive, and human epidermal growth factor receptor 2 (HER2) positive, presented with cutaneous lesions of the upper front body. Skin biopsy revealed infiltration of immature blastoid cells, positive for CD7, CD5, CD10, TdT, CD38, cytoplasmatic CD3 but negative for surface CD3, CD34, MPO, CD8, with a proliferation index of 80‐90%. Subsequent bone marrow (BM) examination identified a cell population with the same phenotype. Fluorescence in situ hybridization identified a *KMT2A* rearrangement, other than AFF1. According to the WHO 2016 Classification, the case was categorized as precursor T‐cell acute lymphoblastic leukemia (T‐ALL). Specific treatment following the German Multicenter Study Group on Adult Acute Lymphoblastic Leukemia (GMALL) 07/03 protocol was initiated [1]. The patient reached complete hematological remission (hCR) but remained positive for measurable residual disease (MRD) (real‐time quantitative polymerase chain reaction (qRT‐PCR), detection limit of 1 × 10^−5^ for both markers). According to protocol, after first consolidation, the patient proceeded to human leukocyte antigen matched related donor allogeneic hematopoietic stem cell transplantation, achieved molecular complete remission and complete resolution of the skin lesions.

**FIGURE 1:**
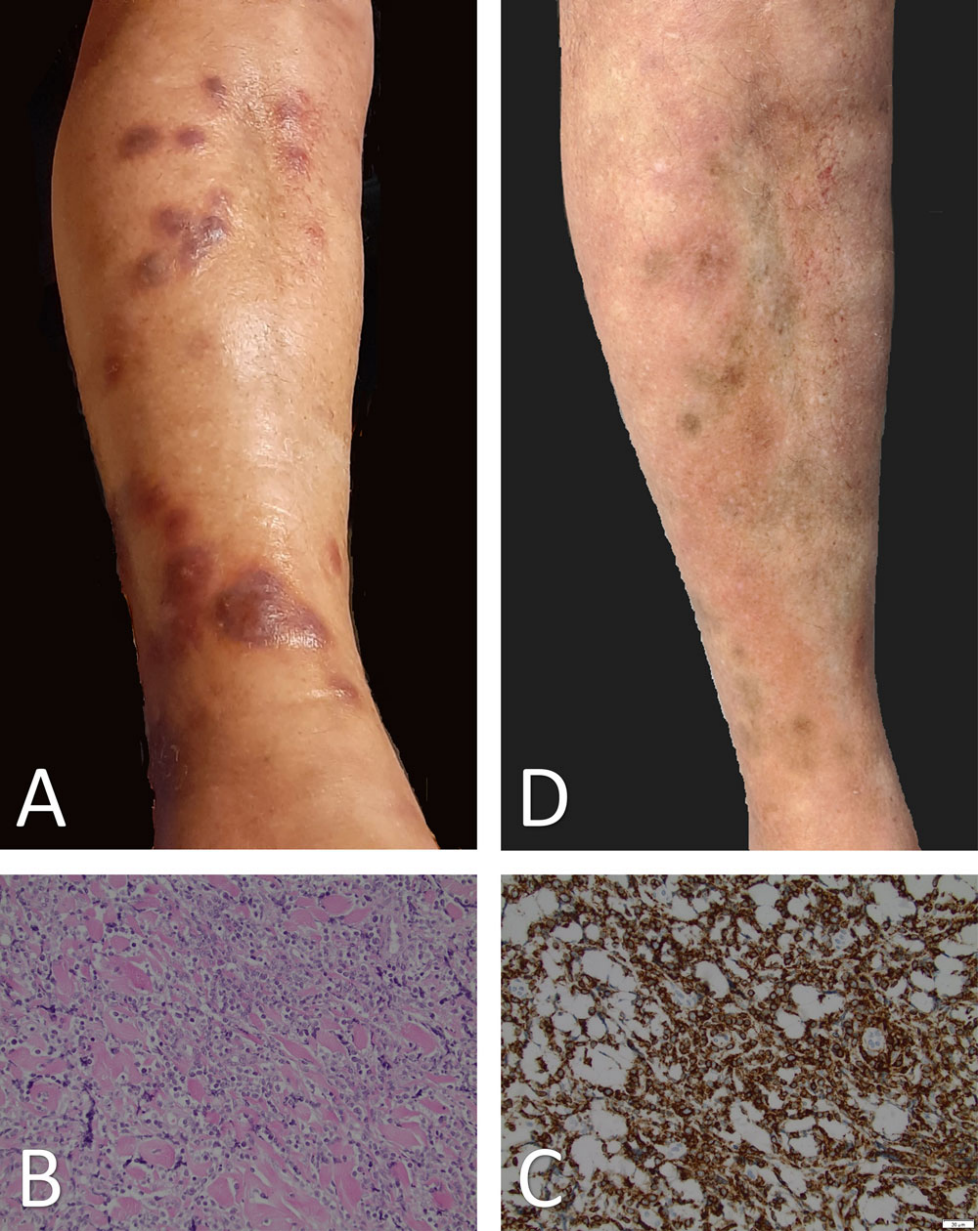
(A) Cutaneous lesions right leg before treatment largest nodule 2 cm × 2 cm in diameter and (B) skin biopsy showing diffuse dermal infiltrates of small, monotonous blastoid cells, sparing the epidermis; HE, original magnification: 200×. (C) Immunohistochemistry of cutaneous lesions showing CD5 positivity of neoplastic infiltrates; original magnification: 200×. (D) Clinical control after 15 days of treatment

In the following years, the patient suffered a series of cutaneous relapses while maintaining complete donor chimerism and MRD negativity until May 2018. After that, the MRD results turned positive at the above‐mentioned detection level while maintaining hCR. Cutaneous relapses were treated as shown in Table 1. The last cutaneous relapse occurred on the right leg (Figure 1A) in March 2020, as confirmed by histology and immunohistochemistry (Figures 1B and 1C). The patient was enrolled in the Molecularly Aided Stratification for Tumor Eradication Research (MASTER) program [2], where a whole genome and transcriptome sequencing performed on the skin biopsy identified several molecular alterations among which there were two heterozygous Janus kinase 3 (*JAK3*) mutations: A573V (variant allele frequency (VAF) 53%) and M511I (VAF, 56%). For both mutations, expression was confirmed by RNA sequencing. In addition, the *KMT2A* rearrangement was identified as a *KMT2A‐ELL* gene fusion by RNA sequencing, and other T‐ALL recurrent mutations (*U2AF1* R35L, *NOTCH1* L1678P, and *CCND3* R190fs) were detected. Given the recent clinical evidence regarding the sensitivity of T‐cell prolymphocytic leukemia to *JAK2* inhibition and the preclinical data in transformed lymphocyte cells regarding the inhibition of JAK1/JAK3 using the *JAK* inhibitors ruxolitinib or tofacitinib [3, 4], the decision was made to pursue therapy with ruxolitinib in combination with tofacitinib. Due to major contraindications to tofacitinib in our patient (deep vein thrombosis of the jugular and subclavian vein and heart failure NYHA III), the salvage therapy with ruxolitinib 10 mg twice daily was initiated. An interim examination on day 15 revealed moderate anemia (Hb 10.5 g/L) with otherwise no relevant side effects from the therapy. The cutaneous lesions completely dissolved (Figure 1D), donor chimerism was 100%, but MRD remained positive at the detection limit (1 × 10^−5^). At the second staging on day 50, we could confirm a complete remission of the skin lesions. No further treatment related toxicities were encountered. Ruxolitinib was discontinued after 5 months due to a cutaneous relapse and increasing MRD in peripheral blood and BM. A therapy with decitabine and venetoclax was started and soon after, due to lack of response, multiple infectious complications and relevant clinical deterioration, treatment deescalation was discussed with the patient and a best supportive care therapy was pursued.

**TABLE 1:** Description of therapies used since diagnosis, best response, and main side effects

Timeline/event	Therapy	Result
January‐April 2015 Initial diagnosis	Prephase, induction, and consolidation therapy based on GMALL 07/03‐Protocol including prophylactic cranial irradiation. [1]	Complete regression of cutaneous lesions, bone marrow puncture: hematological CR, MRD positive
June 2015	Related donor‐allogenic, HLA‐matched blood stem cell transplantation (conditioning: TBI 8 Gy/fludarabine, immunosuppression with CsA/MMF)	Hematological CR, MRD negative (<5 × 10^−4^), bone marrow chimerism: 98‐100% donor
May 2016 and September 2016: 1st and 2nd cutaneous relapse (Hematological CR, MRD positive^a^, bone marrow chimerism: 100% donor)	Radiotherapy (8 × 3 Gy right lower leg,15 × 2 Gy distal right lower leg), DLIs	Hematological CR, MRD negative, bone marrow chimerism: 100% donor Complete dissolution of skin lesions, postradiogenic alterations
April 2017, November 2017, May 2018: 3rd, 4th, 5th cutaneous relapse (hematological CR, MRD negative^a^, bone marrow chimerism: 100% donor)	Radiotherapy (15 × 2 Gy right upper leg, 8 × 3 Gy right lower leg, 15 × 2 Gy abdomen)	Hematological CR, MRD negative, bone marrow chimerism: 100% donor Complete dissolution of skin lesions
November 2018: 6th cutaneous relapse (hematological CR, MRD borderline positive^a^, bone marrow chimerism: 100% donor)	Radiotherapy (15 × 2 Gy abdomen)	Hematological CR, MRD borderline, bone marrow chimerism: 100% donor Complete dissolution of skin lesions
March 2019: 7th cutaneous relapse (PB: MRD borderline positive^a^)	3 cycles of nelarabine	MRD PB: borderline complete dissolution of skin lesions
December 2019: 8th cutaneous relapse (PB: MRD borderline positive^a^)	3 cycles of nelarabine/cyclosphosphamide	MRD PB: negative, regressive cutaneous lesions
March 2020: 9th cutaneous relapse (PB: MRD borderline positive^a^)	Ruxolitinib10 mg twice daily	MRD PB: borderline Complete dissolution of skin lesions, moderate anemia (Hb: 10.5 g/dL)

Abbreviations: CR: complete remission; CsA: cyclosporine; DLI: donor lymphocyte infusion.; Gy: gray; HLA: human leukocyte antigen; JAK: Janus kinase; MMF: mycophenolate mofetil; MRD: measurable residual disease; PB: peripheral blood; T‐ALL: T‐cell acute lymphoblastic leukemia; TBI: total body irradiation.

^a^ Detection limit 1 × 10^−5^.

## 2 DISCUSSION

T‐ALL is a rare, aggressive T‐cell malignancy. Chemotherapy alone cures only 25‐45% of the cases, and despite improvements in the understanding of the origin of the disease, survival remains dismal, with only 50% alive at 5 years [5]. Thus, novel treatment agents and strategies are urgently needed.

Comprehensive molecular analyses have already identified a large number of T‐ALL‐specific oncogenes [6]. Still, the genetic defects underlying the malignant proliferation and survival of the leukemic cells remain clinically unexploited. Recent studies showed that most cases of T‐ALL present with recurrent chromosomal aberrations or mutations [7]. Thus, molecular screening in diagnostic and relapse setting may open‐up additional treatment options. The most frequent genomic alterations in T‐ALL affect PI3K/AKT/mTOR, JAK/STAT, RAF/MEK/ERK, or *NOTCH1* signaling pathways [7].

The JAK/STAT signaling pathway plays an essential role in normal hematopoiesis. *JAK2* helps in the maintenance of hematopoietic stem cells and various stages of myelopoiesis [8], whereas *JAK1* cooperates with *JAK3* for lymphopoiesis. *JAK3* mutations have been reported in 10‐16% of T‐ALL patients, with *JAK3* M511I being the most frequently identified mutation [9]. *JAK3* mutations have been reported to be dependent on binding to the common γ chain of cytokine receptor complexes [10].

Targeted therapy with tofacitinib, a pan‐JAK inhibitor, in combination with ruxolitinib was suggested based on the sequencing results. This was supported by previous preclinical data regarding its effectiveness of targeting *JAK3* mutations as well as a successful therapy with tofacitinib described in a single‐patient case report with T‐ALL and a *JAK3* mutation [11, 12]. Accordingly, tofacitinib, combined with ruxolitinib, has an enhanced potency in vitro in other disease models aiming to inhibit the IL2RG‐JAK1‐JAK3‐STAT5B signaling pathway [13]. As *JAK3* mutants are dependent on *JAK1* signaling for their cellular transformation, it is possible to use both JAK1/JAK2 and JAK3‐selective inhibitors in *JAK3* mutation–positive leukemia [11]. However, as previously mentioned, due to existing contraindications to tofacitinib, single‐agent ruxolitinib was initiated.

As seen in previous publications in T‐ALL murine xenograft models, the patient showed a remarkable response of the skin lesions with ruxolitinib, independently of the presence of *JAK1* or *JAK2* mutations.^14^ Inline, JAK3 M511I was found to obtain an increased JAK/STAT signaling by acquiring an additional mutation in the pseudokinase domain, which was also present in our patient.^15^

Since our patient had complete donor chimerism, we assumed that the graft versus leukemia effect was sufficient to maintain an hCR in the BM but not to prevent the skin relapse. Leukemic cells were able to evade immune surveillance and migrate into peripheral tissues; however, single‐agent ruxolitinib was able to induce complete remission of the extramedullary relapse in our case.

Our results illustrate the potential benefit of the JAK inhibitor ruxolitinib in *JAK3*‐mutated refractory T‐ALL and emphasize the importance of integrating molecular markers in current treatment decision making for patients with T‐ALL. Further exploration of this targeted therapeutic strategy in molecularly stratified patient cohorts is warranted.

## AUTHOR CONTRIBUTIONS

SJ, HH, SS, CMT, and RFS were involved in the diagnosis and treatment of the patient. SJ, HH, and RFS designed the case report. PH, VT, CH, and SF did and interpreted molecular analyses. SF, PH, VT, and CH analyzed the NGS results and reviewed the case within the MASTER program. BH performed bioinformatic analysis. AS, HG, and BG performed the histopathological and immunophenotypic analysis. SJ, HH, and RS wrote the first draft of the manuscript. All authors interpreted the data, and all authors read and contributed to the final version of the manuscript.

## CONFLICT OF INTEREST

The authors declare that there is no conflict of interest.
